# ﻿Coleção de Vetores de Tripanosomatídeos (Fiocruz/COLVET) held at the institution Fiocruz Minas in Brazil: diversity of Triatominae (Hemiptera, Reduviidae) and relevance for research, education, and entomological surveillance

**DOI:** 10.3897/zookeys.1074.69700

**Published:** 2021-12-01

**Authors:** Raíssa N. Brito, Rita C. M. Souza, Liléia Diotaitui, Valeria S. Lima, Raquel A. Ferreira

**Affiliations:** 1 Grupo Triatomíneos, Instituto René Rachou, Fundação Oswaldo Cruz – IRR/Fiocruz Minas, Belo Horizonte, Minas Gerais, Brazil Instituto René Rachou Belo Horizonte Brazil; 2 Programa de Pós-Graduação em Medicina Tropical, Núcleo de Medicina Tropical, Universidade de Brasília (UnB), Brasília, Distrito Federal, Brazil Universidade de Brasília Brasília Brazil

**Keywords:** Biological collections, Chagas Disease, curatorial services, public health, triatomines

## Abstract

The Coleção de Vetores de Tripanosomatídeos (Fiocruz/COLVET), Minas Gerais, Brazil, stands out as one of the most important collections of blood-sucking triatomines, the vectors of *Trypanosomacruzi* that causes Chagas disease. The aim is to describe the collection and the services it provides to support scientific research, educational activities, and entomological surveillance between 2013–2019.The data associated with the specimens held in Fiocruz/COLVET is available from the Sistema de Informação sobre a Biodiversidade Brasileira (SiBBr). These specimen metadata were analyzed and either tabulated or plotted on graph and maps. The records of services provided by the collection between 2013–2019 were also categorized and analyzed. There are 12,568 triatomine specimens deposited in the collection that belong to 77 species and 11 genera, from 15 American countries. Of the ~ 65 species of triatomines found in Brazil, 38 (57.6%) are present in the collection, including specimens from all biomes and all but three Brazilian states. The occurrence of *Triatomacostalimai*, *Triatomalenti*, *Rhodniusnasutus*, and *Panstrongyluslenti* apparently collected beyond their known distribution ranges are reported and discussed. The collection provided 168 services, supporting educational activities (41.7%), scientific research (35.7%), and regional/national entomological surveillance of triatomines (22.6%). Between the years 2014 and 2020, the number of biological specimens deposited in the Fiocruz/COLVET repository increased from 4,778 to 12,568 triatomine specimens. In addition to its great value to biodiversity conservation, the collection is of great importance because of its support of research and educational activities, and contributions to entomological surveillance, and, therefore, to public health.

## ﻿Introduction

Biological collections are organized repositories for ex situ preservation of biological material (Martins 1994; International Commission on Zoological Nomenclature 1999). Attached to the biological material is provenance data associated with each specimen, which includes taxonomic identification, the original date of collection, the name(s) of its collectors, and other relevant data (Martins 1994). This wealth of specimens and their metadata are powerful research tools for basic and applied science, ranging from agriculture to conservation biology, and knowledge of biodiversity to public health (Suarez and Tsutsui 2004; Bakker et al. 2020). In addition to supporting research activities in different areas, such collections have stood out as tools capable of benefiting other segments of society. The so-called ‘outer museum’ or ‘educational collection’ makes the collection/museum materials available for educational purposes, such as practical classes and public exhibits in order to popularize scientific research to all audiences (Allmon 1994; Martins 1994; Bakker et al. 2020). Service provision, such as the correct taxonomic identification of specimens for scientists, professors, farmers, the lay public, and health surveillance staff is another strength of some collections (Allmon 1994; Martins 1994).

Biological collections of medically important organisms contribute to research and many activities that may have implications for public health (Martins 1994; Suarez and Tsutsui 2004; Bakker et al. 2020). Among these are collections of blood-sucking triatomine bugs (Hemiptera: Reduviidae: Triatominae), vectors of *Trypanosomacruzi* Chagas, the parasite that causes Chagas disease, an illness with the highest socio-economic burden in Latin America (Chagas 1909; Kyu et al. 2018). In Brazil, there are three triatomine-specific collections with publicly available catalogues: the Coleção de Vetores de Tripanosomatídeos (Fiocruz/COLVET, http://colvet.fiocruz.br/) at the Instituto René Rachou / Fundação Oswaldo Cruz (IRR/Fiocruz), the Coleção de Triatomíneos do Instituto Oswaldo (Fiocruz/CTIOC, http://ctioc.fiocruz.br/), and the Coleção de Triatominae (https://www2.fcfar.unesp.br/#!/triatominae) at the Faculdade de Ciências Farmacêuticas/Universidade Estadual Paulista Júlio de Mesquita Filho (FCFAR/Unesp). Outside Brazil, there is the Colección de Triatominae of the Mueseo del Instituto de Zoología Agrícola Francisco Fernández Yépez in Venezuela, which houses more than one thousand triatomine specimens (Oliveira et al. 2020). Other collections house important type specimens of Triatominae, among them: the collection of the Museo Argentino de Ciencias Naturales (Bachmann 2012) and the collection of the División Entomología in the Museo de La Plata, both in Argentina (Coscarón et al. 2015); the Hemimetabola Collection of the Museum für Naturkunde, in Berlin, Germany (Rodrigues et al. 2020); and the Smithsonian National Museum of Natural History and the American Museum of Natural History, both in the United States of America.

Fiocruz/COLVET, formerly called Coleção de Vetores da Doença de Chagas (Fiocruz/COLVEC) until 2020, was established in 1996 in the Grupo Triatomíneos (Triatomines Research Group) at the IRR/Fiocruz. The collection is one of the 33 biological collections held at the different research institutes comprising Fiocruz that are officially recognized as an institutional collection: that is, a biological repository that provides services for the academic community and the general public, including the depositing and loaning of specimens, the taxonomic identification of specimens, as well as the training of human resources and the promotion of activities to popularize scientific research for lay audiences (Silva et al. 2020). Between 2006–2009, Fiocruz created a state-of-the-art strategy to support its biological collections. This created not only organizational improvements for its collections, by providing clear guidelines and promoting institutional responsibilities for all of its 33 collections, but also guarantees a steady flow of funding for the maintenance of the collections (Silva et al. 2020). Currently, Fiocruz/COLVET, and other Fiocruz collections, each maintain their own web page (e.g., http://colvet.fiocruz.br/), which provides access to the data associated with the collection specimens via the Sistema de Informação sobre a Biodiversidade Brasileira (SiBBr, https://www.sibbr.gov.br/) and Global Biodiversity Information Facility (GBIF, https://www.gbif.org/). The maintenance of the online catalogues by the collections is an advance, since it makes available information associated with the specimens to the public (Cook et al. 2014). The implementation of the Quality Management System at Fiocruz/COLVET through the regulation NBR ISO/IEC 17025 was an important step to improve services provided by the collection (Silva et al. 2020), as compliance with this regulation guarantees the quality of the information and services provided by Fiocruz/COLVET.

Fiocruz/COLVET comprises two repositories: (i) scientific and (ii) educational, that house triatomine specimens for different purposes. The scientific collection supports research regarding the biogeography, ecology, genetics, evolution, systematics, and taxonomy of triatomines. On the other hand, the educational repository supports educational activities for academic purposes and the general public. In addition, both scientific and educational repositories contribute to routine entomological surveillance services. In 2014, an article was published presenting the diversity of triatomines deposited in the scientific repository of Fiocruz/COLVET (Souza et al. 2014). However, the number of specimens deposited in the scientific collection has been increasing and, consequently, the diversity of species as well. Moreover, the collection underwent an internal restructuring, involving the implementation of updated quality management standards, reviewing, and expanding its standard operating procedures (SOPs), and reviewing and reorganizing its physical and online databases. Thus, here we aim to publish an update on the number of triatomine specimens deposited in Fiocruz/COLVET and describe their most relevant associated information. We also present the first compilation of services provided by the collection since 2013. Finally, we discuss the relevance of Fiocruz/COLVET in the light of its benefits for science, society, and public health.

## ﻿Materials and methods

### ﻿Coleção de Vetores de Tripanosomatídeos (Fiocruz/COLVET)

The scientific repository of Fiocruz/COLVET houses hemipteran specimens, mainly Triatominae, that are organized into four different sections:

(I) Whole and ‘semi-whole’: whole intact adult/nymph hemipteran specimens and semi-whole adult/nymph hemipteran specimens comprising the head, thorax, and abdomen, but with the wings, legs and/or external genitalia removed. Adults (males and females), and fourth- and fifth-stage nymphs, are preserved dry inside steel cabinet drawers closed with a clear acrylic cover. These specimens are pinned through the thorax with an entomological pin. The first- to third-stage nymphs are preserved dry as double mounts (i.e., mounted on a card point, which in turn is attached to an entomological pin).

(II) Partial triatomine specimens: separate triatomine body parts, such as heads, abdomens, legs, and external genitalia, which are preserved dry, or in 70% ethanol, and individually stored in polypropylene microtubes. Wings are preserved dry and stored (each with its respective pair) in polypropylene microtubes or fixed between two microscope slides. In general, these body parts were separated from their associated body to perform taxonomical and ecological studies and were then subsequently deposited in the collection.

(III) Eggs: preserved dry as double mounts.

(IV) Triatomine DNA: genomic extracts stored in polypropylene microtubes, cryopreserved in liquid nitrogen, at -196°C.

Each specimen deposited in the collection receives a label containing a catalogue number. In Section III above, the specimens are independent and, therefore, each has its own unique catalog number. However, in Sections I, II, and IV, there may be different body parts from the same individual triatomine in more than one section. In these cases, the catalogue number of each part from the same individual triatomine (in Sections II and IV) will be the same as the corresponding semi-whole specimen (in Section I), followed by the first letter of the corresponding part of the triatomine. It is important to note that in Section II, there are also body parts that are independent specimens, since the other body sections of the corresponding triatomine were not deposited in the collection. In these latter cases, the specimen parts have their own unique catalogue number followed by the first letter of the corresponding body part. This means that the total number of specimen records in the collection is greater than the total number of individual triatomines held.

The biological material deposited in Fiocruz/COLVET comes from three main sources: (1) ‘research voucher specimens’, whole (or semi-whole) triatomines, their body parts and/or DNA samples, that were used to perform scientific research and then deposited in the collection; (2) whole or parts of triatomines and eggs reared in insectaries (i.e., laboratory stocks), donated from different institutions, or caught in domiciles or sylvatic environments by researchers or others; and (3) triatomines donated by the reference service in taxonomic triatomine identification of the Grupo Triatomíneos at IRR/Fiocruz (Suppl. material 1). This reference service provides support to the routine entomological surveillance for Chagas disease performed by the public health services in Brazil, which is carried out by Brazilian state/regional health departments. The reference service provides support in two ways: (i) via validation of taxonomic identification of triatomines caught by the entomological surveillance services, and (ii) by promoting training in the taxonomic identification of triatomines for the professionals working as part of the Chagas disease control program.

Finally, the educational repository of Fiocruz/COLVET is comprised of specimens that are either partially damaged or with incomplete provenance, as well as specimens whose number of individuals in the scientific repository is already representative of several populations within the ranges of the species distribution (Suppl. material 1).

### ﻿Fiocruz/COLVET metadata analysis

The Fiocruz/COLVET database is available from SiBBr (https://ala-hub.sibbr.gov.br/ala-hub/occurrences/search?q=collection_uid:co52) in the Darwin Core Archive (DwC-A) file format. This zip file contains text files that can be read by different types of software. We used Microsoft Excel to tabulate the data (e.g., species, genus, country, state and site of capture, etc.) and to plot them as graph and tables. Maps showing the geographical distribution of species were created using Quantum GIS 3.18.2 (QGIS, https://qgis.org). The total number of species present in the educational repository was visually quantified.

The following data were analyzed: (i) the total number of specimens in the collection; (ii), the number of body parts from the same individuals, showing the amount of biological material available according to the deposited taxa; (iii) the diversity of triatomines from Brazil present in the collection, according to their biomes and states of origin; (iv) the most epidemiological important triatomine species in Brazil and their representativeness in the collection; (v) the diversity of triatomine species from other American countries; and (vi) the diversity of triatomine species of unknown origin and from laboratory stocks.

### ﻿Services provided by Fiocruz/COLVET

The collection provides services to the scientific and non-scientific community upon request using a specific form available on the collection website (http://colvet.fiocruz.br/). Since 2013, all services provided by the collection are recorded by the curator through these request forms. We used the records of these requests generated between the years 2013–2019 to quantify and classify them into: services provided to support (i) scientific research, (ii) educational activities, and (iii) entomological surveillance. The first category comprises services provided that were requested by researchers for scientific purposes, including (a) depositing in the scientific collection of research voucher specimens used in previous research projects, (b) requests for training in the taxonomic identification of Triatominae, (c) verification of taxonomic identification of Triatominae, (d) loaning or donating specimens from the scientific repository, (e) access to specific specimens from the scientific repository through *in loco* or online consultations, and (f) scientific consultancy for different purposes (e.g., instructions on the use of taxonomic identification tools or of biological collections databases). The services provided to support educational activities include loaning/donating specimens from the educational repository to students or teachers (from secondary to higher education) to be used in practical classes, group work, educational projects, and/or activities to popularize scientific knowledge for lay audiences. Finally, the services provided by the collection to support routine entomological surveillance comprise depositing in the scientific collection of triatomines identified by the reference service and training in triatomine identification for health service workers.

## ﻿Results

The scientific repository of Fiocruz/COLVET contains 13,126 biological records related to insects in general. Of these, 95.7% (12,568) are specimens of the subfamily Triatominae. The remaining 4.3% (558) are specimens from other hemipteran families, as follows: Reduviidae (491), Pentatomidae (55), Coreidae (6), Aradidae (2), and Phloeidae (2). In addition, there are also two hymenopteran specimens belonging to the family Apidae. A summary of all the specimen records of triatomines is shown in Table 1, which lists information on whole/semi-whole triatomines, their parts, eggs, and DNA samples, from individuals that were either caught in sylvatic or domestic/peri-domestic environments, or derived from laboratory stocks, or are of unknown origin. Whole/semi-whole triatomines with known and unknown origin comprise 70.7% (8,889) of the total specimen records related to the subfamily Triatominae whereas 27.2% (3,422) represent body parts, 0.5% (61) are eggs, and 1.6% (196) are DNA samples (Table 1).

**Table 1.** Summary of the records of biological material from triatomine species deposited in the scientific repository of the Coleção de Vetores de Tripanosomatídeos (Fiocruz/COLVET) that either came from sylvatic or domestic/peridomestic environments, laboratory stocks, or are of unknown origin. The records include whole triatomines, body parts, eggs, and cryopreserved DNA.

Species	Section I		Section II	Section III	Section IV	Total specimens	Countries
	Whole/semi–whole of known origin	Whole of unknown origin	Body parts	Eggs	DNA		
**Tribe Triatomini**							
*Dipetalogastermaxima*	6	–	–	–	–	6	1
*Eratyruscuspidatus*	4 (2)	2	–	–	–	8	3
*Eratyrusmucronatus*	29	5	–	5*	–	39	3
*Hermalentiamatsunoi*	1	–	–	–	–	1	1
*Mepraiaspinolai*	1	–	–	–	–	1	1
*Nesotriatomaflavida*	–	33	–	–	–	33	–
*Pantrongyluschinai*	5 (2)	–	–	–	–	7	1
*Pantrongylushumeralis*	1	1	–	–	–	2	1
*Pantrongyluslenti*	1	–	–	–	–	1	1
*Pantrongyluslignarius*	10 (52)	2	–	–	–	64	3
*Pantrongyluslutzi*	158	1	–	–	–	159	1
*Pantrongylusmegistus*	718 (6)	12	1,736^a^	10		2,482	3
*Pantrongylusrufotuberculatus*	10	1	–	–	–	11	2
*Paratriatomahirsuta*	1	–	–	–	–	1	1
*Paratriatomalecticularia*	9	3	–	–	–	12	2
*Triatomabarberi*	5	7	–	–	–	12	2
*Triatomadiasi*	60	6	–	–	–	66	1
*Triatomadimidiata*	37 (4)	5	–	–	–	46	4
*Triatomageniculatus*	71	4	3	–	–	78	4
*Triatomagerstaeckeri*	2	–	–	–	–	2	1
*Triatomalongipennis*	3	–	–	–	–	3	1
*Triatomamaculata*	180	2	–	1	–	183	2
*Triatomamazzotti*	7	–	–	–	–	7	1
*Triatomamelanocephala*	1 (6)	–	(19)^b^	–	–	27	1
*Triatomanigromaculata*	12	–	–	–	–	12	1
*Triatomanitida*	–	1	–	–	–	1	–
*Triatomapallidipennis*	7	–	–	–	–	7	1
*Triatomaphyllosoma*	3	–	–	–	–	3	1
*Triatomapicturata*	35	2	–	–	–	37	1
*Triatomaprotracta*	6	3	–	–	–	9	1
*Triatomarubida*	–	1	–	–	–	1	–
*Triatomarubrofasciata*	16	–	–	–	–	16	1
*Triatomasinaloensis*	–	1	–	–	–	1	–
*Triatomatibiamaculata*	1 (2)	4	–	–	–	7	1
*Triatomavitticeps*	357 (97)	1	103^c^	7 (17)	–	582	1
*Tritomaarthurneivai*	28	4	–	–	–	32	1
*Tritomabaratai*	4	3	–	–	–	7	1
*Tritomabrasiliensis*	639 (8)	219	270	–	156	1,292	1
*Tritomabrasiliensismacromelasoma*	4	–	–	–	–	4	1
*Tritomacarcavalloi*	–1	2	–	–	–	3	–
*Tritomacostalimai*	10 (1)	4	–	–	–	15	1
*Tritomadelpontei*	–	3	–	–	–	3	–
*Tritomagarciabesi*	1	5	–	–	–	6	2
*Tritomaguasayana*	14	12	–	–	–	26	1
*Tritomaguazu*	–	3	–	–	–	3	–
*Tritomainfestans*	803 (11)	7	141^d^	–	–	962	3
*Tritomajuazeirensis*	8	–	–	–	–	8	1
*Tritomalenti*	21 (3)	12	15^e^	–	–	51	1
**Tribe Triatomini**							
*Tritomamatogrossensis*	5	11	–	–	–	16	1
*Tritomamelanica*	33	–	89^f^	–	–	122	1
*Tritomaoliveirai*	–	2	–	–	–	2	–
*Tritomapetrocchiae*	4	2	–	–	–	6	1
*Tritomaplatensis*	–	7	–	–	–	7	–
*Tritomapseudomaculata*	476	5	144	–	–	625	1
*Tritomarubrovaria*	22	4	–	–	–	26	2
*Tritomasordida*	2,871 (16)	28	77 (4)	2	–	2,997	2
*Tritomavandae*	–1	–	–	–	–	1	–
*Tritomawilliami*	10	2	–	–	–	12	1
*Tritomawygodzinskyi*	44	–	–	–	–	44	1
**Tribe Cavernicolini**							
*Cavernicolalenti*	12 (39)	1	–	–	–	52	1
*Cavernicolapilosa*	52 (19)	–	–	(1)	–	72	2
**Tribe Rhodniini**							
*Rhodniuspallescens*	3	3	–	–	–	6	1
*Psammolestesarthuri*	56	5	–	4 (1)	–	66	1
*Psammolestescoreodes*	5	–	–	–	–	5	1
*Psammolestestertius*	20	1	–	–	–	21	1
*Rhodniusbrethesi*	2 (6)	2	–	–	–	10	1
*Rhodniuscolombiensis*	2	–	–	–	–	2	1
*Rhodniusdomesticus*	21	1	–	–	–	22	1
*Rhodniusecuadoriensis*	6	8	–	–	–	14	1
*Rhodniusnasutus*	421 (69)	–	650 (60)^g^	–	–	1,200	1
*Rhodniusneglectus*	421 (2)	37	30	–	8	498	1
*Rhodniusneivai*	10	–	–	13	–	23	1
*Rhodniuspictipes*	45 (5)	6	–	–	–	56	2
*Rhodniusprolixus*	86 (2)	1	–	–	–	89	1
*Rhodniusrobustus*	109	–	81^h^	–	32	222	4
*Rhodniusstali*	10	–	–	–	–	10	1
**Non-identified**							
*Psammolestes* sp.	1	–	–	–	–	1	1
*Rhodnius* sp.	1	–	–	–	–	1	1
Triatominae	1	–	–	–	–	1	1
**Total**	**8,392**	**497**	**3,422**	**61**	**196**	**12,568**	

For the ‘whole’, ‘semi-whole’, ‘body parts’, ‘eggs’ and ‘DNA’ columns: the numbers within parenthesis indicate specimens from laboratory stocks (i.e., reared in insectaries), whereas numbers outside the parenthesis indicate the number of specimens caught in sylvatic or domestic/peridomestic environments in the field. * Indicates that 1 egg of *Eratyrusmucronatus* is from an unknown origin. ^a^Among the 1,736 body parts of *Panstrongylusmegistus*, 1,715 are independent specimens (i.e., body parts not associated with either a semi-whole triatomine or other body parts or DNA), 20 body parts are non-independent specimens (i.e., two body parts from each of 10 different individuals for which there is no associated semi-whole specimen), and one is a body part from a single semi-whole individual. ^b^Among the 19 body parts of *Triatomamelanocephala*, 18 are independent specimens, and one is a non-independent specimen of one semi-whole individual. ^c^Among the 103 body parts of *Triatomavitticeps*, 73 are independent specimens, 30 are non-independent specimens from 30 semi-whole individuals. ^d^Among the 141 body parts of *Triatomainfestans*, one is an independent specimen, and 140 are non-independent specimens (i.e., two body parts from each of 70 different individuals for which there is no associated semi-whole specimen). ^e^All 15 body parts of *T.lenti* are non-independent specimens from 15 semi-whole individuals. ^f^Among the 89 body parts of *Triatomamelanica*, 3 are independent specimens, and 86 are non-independent specimens (i.e., two body parts from each of different 43 individuals for which there is no associated semi-whole specimen). ^g^Among the 710 body parts of *Rhodniusnasutus*, 304 are independent specimens, and 406 are non-independent specimens (i.e., two body parts from each of 203 different individuals for which there is no associated semi-whole specimen). ^h^Among the 81 body parts of *Rhodniusrobustus*, 35 are independent specimens, and 46 are non-independent specimens (i.e., two body parts from each of 23 different individuals for which there is no associated semi-whole specimen).

There is currently a total of 12,172 triatomine individuals deposited in the scientific repository and 558 individuals from the other families mentioned above. Regarding the number of species, the collection houses 77 Triatominae species from 11 genera.

### ﻿Diversity in the Fiocruz/COLVET scientific repository of Triatominae collected in Brazil

#### Section I – Dry-preserved whole/semi-whole individuals

**Figure 1.**
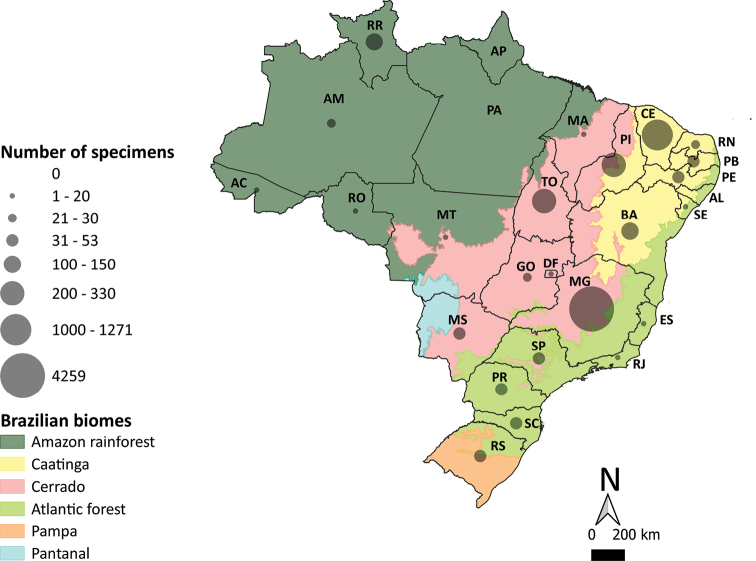
Summary of the whole/semi-whole triatomine specimens according to Brazilian state deposited in the Coleção de Vetores de Tripanosomatídeos (Ficoruz/COLVET). The Brazilian states are abbreviated as follows: (AC) Acre, (AL) Alagoas, (AP) Amapá, (AM) Amazonas, (BA) Bahia, (CE) Ceará, (DF) Distrito Federal, (ES) Espírito Santo, (GO) Goiás, (MA) Maranhão, (MT) Mato Grosso, (MS) Mato Grosso do Sul, (MG) Minas Gerais, (PA) Pará, (PB) Paraíba, (PR) Paraná, (PE) Pernambuco, (PI) Piauí, (RJ) Rio de Janeiro, (RN) Rio Grande do Norte, (RS) Rio Grande do Sul, (RO) Rondônia, (RR) Roraima, (SC) Santa Catarina, (SP) São Paulo, (SE) Sergipe, and (TO) Tocantins.

In total, in the Fiocruz/COLVET scientific repository, there are 6,788 whole/semi-whole triatomine individuals caught in sylvatic or domestic/peri-domestic environments from Brazil. These specimens comprise 38 triatomine species caught in 24 of the 27 Brazilian states (Table 2). The states of Minas Gerais, followed by Ceará, Tocantins, Piauí and Bahia contribute the greatest numbers of triatomine specimens and species deposited in the scientific repository. More than 4,000 specimens from 18 species from the state of Minas Gerais are deposited in the collection. A total of 12 species from the state of Bahia, nine from Tocantins, and eight from Ceará is also represented in the collection. In contrast, the states of Alagoas, Amapá and Pará are underrepresented (Fig. 1, Table 2).

**Table 2.** Summary of the whole/semi-whole triatomine specimens caught in either sylvatic or domestic/peridomestic environments deposited in the Coleção de Vetores de Tripanosomatídeos (Fiocruz/COLVET) according to their capture location within Brazil.

Species	Brazilian states																										
	AC	AL	AP	AM	BA	CE	DF	ES	GO	MA	MT	MS	MG	PA	PB	PR	PE	PI	RJ	RN	RS	RO	RR	SC	SP	SE	TO
*C.lenti*				12																							
*C.pilosa*													5														42
*E.mucronatus*				6																							
*P.diasi*													60														
*P.geniculatus*				4	1	3					2		50														4
*P.lenti*																											**1**
*P.lignarius*				6																							
*P.lutzi*						121							32				3			2							
*P.megistus*					17	26	3		4				616			2								29			
*Ps.arthuri*																											
*Ps.tertius*						5							5							2							8
*R.brethesi*				2																							
*R.domesticus*													1											20			
*R.nasutus*					9	377							**30**					3		2							
*R.neglectus*					2				15	1			184												3		216
*R.pictipes*										1																	26
*R.robustus*	2																					15	8				31
*T.arthurneivai*													28														
*T.b.macromelasoma*																		4									
*T.baratai*												4															
*T.brasiliensis*					6	383									42		33	159		15						1	
*T.costalimai*							1						**9**														
*T.infestans*					8				3				23			31					41				3		
*T.juazeirensis*					8																						
*T.lenti*					1								**20**														
*T.maculata*																							139				
*T.matrogrossensis*											5																
*T.melanica*													33														
*T.melanocephala*																	1										
*T.petrocchiae*																				4							
*T.pseudomaculata*					17	347							38		11		1	59		2							1
*T.rubrofasciata*						9				2							4		1								
*T.rubrovaria*																					12						
*T.sordida*					52						4	20	2,769														1
*T.tibiamaculata*																									1		
*T.vitticeps*					1			3					353														
*T.williami*											3	7															
*T.wygodzinsky*													2												42		
*Rhodnius* sp.					1																						
Triatominae non-identified													1														
Total number of specimens	2	0	0	30	123	1,271	4	3	22	4	14	31	4,259	0	53	33	42	225	1	27	53	15	147	49	49	1	330
Total number of species	1	0	0	5	12	8	2	1	3	3	4	2	18	0	2	2	5	4	1	6	2	1	2	2	4	1	9

Brazilian states are abbreviated as follows: (AC) Acre, (AL) Alagoas, (AP) Amapá, (AM) Amazonas, (BA) Bahia, (CE) Ceará, (DF) Distrito Federal, (ES) Espírito Santo, (GO) Goiás, (MA) Maranhão, (MT) Mato Grosso, (MS) Mato Grosso do Sul, (MG) Minas Gerais, (PA) Pará, (PB) Paraíba, (PR) Paraná, (PE) Pernambuco, (PI) Piauí, (RJ) Rio de Janeiro, (RN) Rio Grande do Norte, (RS) Rio Grande do Sul, (RO) Rondônia, (RR) Roraima, (SC) Santa Catarina, (SP) São Paulo, (SE) Sergipe, and (TO) Tocantins. In **bold**: specimen(s) for which the species was not previously reported to occur in that state, or that were previously reported by others, but the distribution limits were not discussed.

The collection houses specimens native to all Brazilian biomes, e.g., *Rhodniusrobustus* Larrousse from the Amazon rainforest; *Triatomavitticeps* Stål and *Panstrongylusmegistus* Burmeister from the Atlantic forest; *Triatomabaratai* Carcavallo & Jurberg from the seasonally-flooded lowland Pantanal; and *Triatomabrasiliensis* Neiva, *Triatomasordida* Stål and *Triatomarubrovaria* Blachard that are native to the dry, open lowlands of the Caatinga, Cerrado and Pampas, respectively (Fig. 1, Table 2).

**Figure 2.**
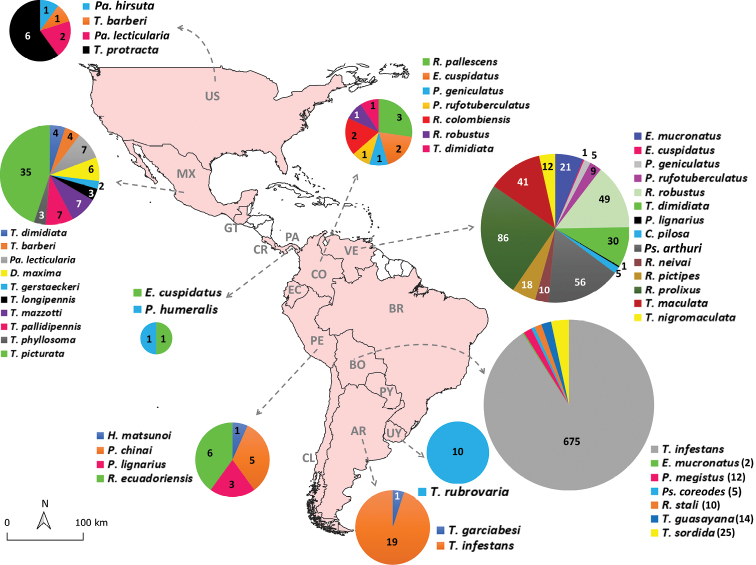
Summary of the specimens caught in sylvatic and domestic/peri-domestic environments deposited in the Coleção de Vetores de Tripanosomatídeos (Fiocruz/COLVET) according to species and location (North, Central, and South American countries). Countries colored pale pink have at least one specimen deposited in the collection. Countries are abbreviated as follows: (US) United States, (MX) Mexico, (GT) Guatemala, (CR) Costa Rica, (PA) Panama, (VE) Venezuela, (CO) Colombia, (EC) Ecuador, (PE) Peru, (BO) Bolivia, (CL), Chile, (PY) Paraguay, (AR) Argentina, and (UY) Uruguay. Also, nine specimens of *P.megistus* from Paraguay, three specimens of *Rhodniusrobustus* from Ecuador, two of *T.dimidiata* from Guatemala, and one specimen, respectively, of *Pantrongylusgeniculatus* and *Mepraiaspinolai* from Costa Rica and Chile are deposited in the scientific repository Ficoruz/COLVET. One specimen morphologically identified as a *Psammolestes* sp. and caught in Venezuela is also deposited in the collection.

The five species with greatest medical relevance in Brazil (i.e., *Triatomainfestans* Klug, *P.megistus*, *T.sordida*, *T.brasiliensis*, and *Triatomapseudomaculata* Corrêa & Spínola are well-represented in the scientific repository, both in terms of the numbers of specimens and populations, and/or sample origin. Although *T.infestans* is not native to Brazil, it was the main vector of *T.cruzi* in the country, and was virtually eliminated from Brazilian domiciles through residual insecticide spraying campaigns (Abad-Franch et al. 2013). The scientific repository houses 109 whole/semi-whole specimens of *T.infestans* that were caught in domiciles from six Brazilian states (Table 2). The 697 whole/semi-whole *P.megistus* individuals come from sylvatic and domestic/peri-domestic environments of seven Brazilian states encompassing the moist Atlantic forests and the dry, open Caatinga and Cerrado biomes (Fig. 1, Table 2). *Triatomasordida* accounts for the greatest number of whole/semi-whole specimens (2,846) from Brazil deposited in the scientific repository. All of these individuals came from five states within the Cerrado biome. *Triatomabrasiliensis* has 639 whole/semi-whole specimens, which are from seven states in the semi-arid Caatinga of north-eastern Brazil (Fig. 1, Table 2). Finally, *T.pseudomaculata* is represented by 476 whole/semi-whole specimens from eight states in north-eastern, south-eastern, and southern Brazil (Table 2).

Fifty-nine adult individuals morphologically-identified as *Triatomacostalimai* Verano & Galvão (9), *Triatomalenti* Sherlock & Serafim (20), and *Rhodniusnasutus* Stål (30) from the northern municipalities of the state of Minas Gerais are deposited in the scientific repository (see Table 2). However, these species are not otherwise recorded as occurring in Minas Gerais. Whether these species really are present in the state of Minas Gerais, outside the known range of their distribution, is discussed further below. The unique *Panstrongyluslenti* Galvão & Palma specimen deposited in Fiocruz/COLVET came from the state of Tocantins (Table 2), but until now, this species has only been reported in the states of Bahia and Goiás.

#### Sections II, III, and IV – Body parts, eggs, and DNA samples

All body parts and DNA samples of triatomines deposited in the scientific repository are research voucher specimens from completed scientific projects. These specimens include 3,422 body parts (wings, heads, abdomens, legs, and/or genitalia), and 196 DNA samples (Table 1). The number of eggs of *T.vitticeps*, *T.sordida*, and *P.megistus* from Brazil deposited in the collection is also included in Table 1.

### ﻿Diversity in the Fiocruz/COLVET scientific repository of Triatominae from other American countries

#### Section I – Dry-preserved whole individuals

A total of 1,250 whole triatomine individuals deposited in the Fiocruz/COLVET scientific repository came from other 14 American countries: United States of America (10), Mexico (78), Guatemala (2), Costa Rica (1), Panama (2), Venezuela (345), Colombia (11), Ecuador (3), Peru (15), Bolivia (743), Chile (1), Paraguay (9), Uruguay (10) and Argentina (20). These specimens comprise 40 triatomine species and one triatomine specimen of the genus *Psammolestes* (Fig. 2). The countries with the largest number of triatomine species deposited in the collection are Venezuela (14), Mexico (10), Colombia (7) and Bolivia (7). The United States of America and Peru are represented in the collection by four species, whereas Panama and Argentina are represented by two triatomine species. The remaining countries of Guatemala, Costa Rica, Ecuador, Chile, Paraguay, and Uruguay are represented by only one species deposited in the collection (Fig. 2).

Whole specimens of *Panstrongylusgeniculatus* Latreille and *R.robustus* present in the collection also came from three different American countries, as well as Brazil. Likewise, *Panstrongyluslignarius* Walker, *P.megistus*, *Eratyrusmucronatus* Stål and *T.infestans* also came from two other South American countries besides Brazil. Similarly, specimens of *Triatomamaculata* Erichson, *Cavernicolapilosa* Barber and *Rhodniuspictipes* Stål from Venezuela and specimens of *T.sordida* and *T.rubrovaria* from Bolivia and Paraguay, respectively, are also deposited in Fiocruz/COLVET (Fig. 2, Tables 1, 2).

*Triatomadimidiata* Latreille is well-represented in the collection by 37 specimens that came from North, Central and South American countries: Guatemala, Mexico, Venezuela, and Colombia. Also, *Eratyruscuspidatus* Stål specimens from South and Central American countries, Venezuela and Panama, can be found in the collection (Fig. 2).

The collection also houses the North American species *Paratriatomahirsuta* Barber and *Triatomaprotracta* Uhler, from the United States of America, and *Triatomabarberi* Usinger and *Paratriatomalecticularia* Stål from both the United States of America and Mexico. Other North American species *Triatomagerstaeckeri* Stål, *Triatomalongipennis* Usinger, *Triatomamazzotti* Usinger, *Triatomapallidipennis* Stål, *Triatomaphyllosoma* Burmeister, *Tritomapicturata* Usinger, and *Dipetalogastermaxima* Uhler are represented in the scientific repository by specimens from Mexico (Fig. 2). The North American lineage species *Hermanlentiamatsunoi* Fernández Loayza and *Mepraiaspinolai* Porter from Peru and Chile, respectively, are also represented by only one specimen each (Fig. 2, Table 1).

#### Section IIII – Eggs

In addition to the whole specimens, eggs of *Rhodniusneivai* Lent (13), *T.maculata* (1), *E.mucronatus* (4) and *Psammolestesarthuri* Pinto (4) from Venezuela are also deposited in the collection (Table 1).

### ﻿Specimens from laboratory stocks and of unknown origin in the Fiocruz/COLVET scientific repository

#### Section I – Dry-preserved whole/semi-whole individuals

The scientific repository also houses samples from several laboratory stocks, which have uncertain origins. Table 1 shows that 354 whole/semi-whole specimens are from laboratory stocks. Also, there are 497 specimens of 51 triatomine species without associated information and, hence, of unknown provenance. Among these species, 41 are also represented in the collection by at least one specimen of known provenance and, in some cases, also by specimens from laboratory stocks as well (Table 1).

### ﻿The educational collection

The educational repository comprises 51 triatomine species of the genus *Triatoma*, *Dipetalogaster*, *Panstrongylus*, *Eratyrus*, *Rhodnius*, *Cavernicola*, and *Psammolestes* (Suppl. material 2). Additionally, the educational repository includes specimens that are either phytophagous or predators. Suppl. material 2 lists the species present in the educational repository that are used for educational purposes and support entomological surveillance.

### ﻿Services provided by the educational and scientific repositories of Fiocruz/COLVET

**Figure 3.**
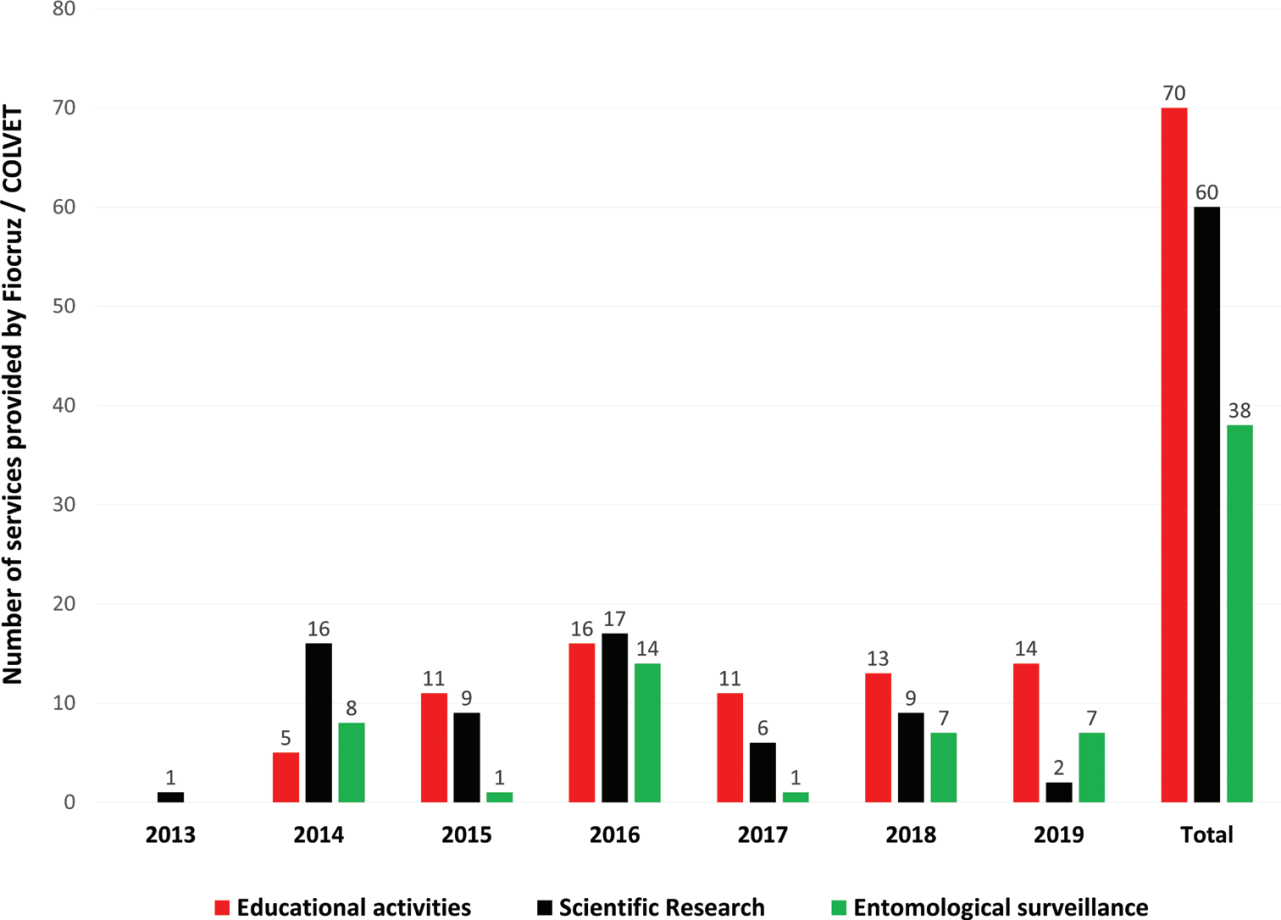
Total number of services provided by the Coleção de Vetores de Tripanosomatídeos (Fiocruz / COLVET) between 2013-2019. The services included were for scientific research, entomological surveillance and educational activities as described in the *Methods* section of the main text.

Between 2013 and 2019, Fiocruz/COLVET provided 168 service requests (Fig. 3). The average number of service requests was 24 per year. In 2016, there were 47 requests, followed by 29 requests answered in each of the years 2014 and 2018. Twenty-three requests were finished by the collection in 2019, 21 during the year 2015 and 18 in 2017. There was only one service provided by the collection in 2013.

Regarding the type of services provided by Fiocruz/COLVET, 41.7% (70) of the total were provided to support educational activities, whereas 35.7% (60) and 22.6% (38) were to support scientific research and routine entomological surveillance, respectively (Fig. 3). Among the 70 services provided for educational purposes, 68 were requests for the donation of specimens from the educational repository for use in practical classes by teachers (from secondary school to higher education) or for production of entomological boxes by undergraduate students. The other two requests were for the loan of specimens from the educational repository for use in public exhibitions to popularize scientific research to lay audiences. The requesters were from 14 Brazilian institutions: Universidade Federal do Rio de Janeiro (UFRJ), Universidade Federal de Viçosa (UFV), Universidade Federal de Minas Gerais (UFMG), Universidade Federal de Juiz de Fora (UFJF), Universidade Federal do Tocantins (UFTO), Universidade Federal de Ouro Preto (UFOP), Universidade Federal de Rondônia (UNIR), Universidade do Estado de Minas Gerais (UEMG), Centro Universitário de Belo Horizonte (Uni-BH), Pontifícia Universidade Católica de Minas Gerais (PUC/MG), Universidade José do Rosário Vellano (UNIFENAS), Faculdade de Minas (FAMINAS-BH), Instituto Metodista Izabela Hendrix, and Instituto René Rachou (IRR / Fiocruz). In addition to these institutions, requesters from secondary (3), primary (1) and technical (1) schools in the state of Minas Gerais also requested services from the collection for educational purposes.

Among the 60 service requests to support scientific research, 36 were deposits of research voucher specimens used in research projects, eight were consultations of the scientific collection by researchers, three were requests for verification of taxonomic identification, six were requests for training in taxonomic identification of triatomines, three were for the loan of specimens, and two were donations of legs from the scientific repository. The remaining two services were technical advice regarding deposited specimens and the usage of the online database of collections. All services provided to support scientific research were for researchers from several institutes, including Brazilian state/regional health departments and universities (e.g., Secretaria de Estado da Saúde de São Paulo, Universidade Federal do Piauí [UFPI], Instituto Metodista Izabela Hendrix, Universidade de Brasília [UnB], UFOP, Universidade Federal do Vale do Jequitinhonha e Mucuri [UFVJM], Fiocruz of Rondônia, IRR / Fiocruz, IOC/ Fiocruz and the Brazilian Ministry of Health) and non-Brazilian universities (e.g., London School of Hygiene and Tropical Medicine, Universidad de Valencia in Spain, Universidad Nacional Autónoma de Mexico, and Centro Regional de Investigaciones Científicas y Transferencia Tecnológica de La Rioja [Crilar] and Universidad de La Plata, both in Argentina).

Among the 38 service requests that supported actions related to entomological surveillance, 16 were deposits of specimens into the scientific repository, 12 were donations of specimens from the educational collection, and ten were requests for the training of technicians in the taxonomic identification of triatomines. The requesting institutions included the reference service for identification of triatomines of the IRR/Fiocruz, the special indigenous health department (Secretaria Especial de Saúde Indígena, SESAI), the state health departments of Ceará, Minas Gerais and Rondonia, and other regional institutions of the states of Minas Gerais and Ceará.

## ﻿Discussion

In the last seven years, the scientific repository of Fiocruz/COLVET went through an increase in deposits of biological material, jumping from 4,778 specimens of triatomines in the year 2014 (Souza et al. 2014) to 12,568 in the year 2020. Accordingly, there was also an increase in the number of species and specimens, i.e., species not yet represented in the scientific repository and specimens with provenance from different locations (i.e., municipalities, states, countries, biomes, and ecotopes). This increase has also expanded the population variability of many species, especially *T.sordida, T.vitticeps*, *T.brasiliensis*, and *P.megistus*.

In 2014, the scientific repository housed 56 species of triatomines (Souza et al. 2014). Presently, there are 77 species deposited in the scientific repository. This total represents almost half of the ~ 150 species of triatomines currently recognized (Dorn et al. 2018; Monteiro et al. 2018; Lima-Cordón et al. 2019; Alevi et al. 2020; Zhao et al. 2021). Since 2014, a great increase in the collection was of the North American species lineage, many of which had no specimens previously deposited in the collection. Regarding the South American species, there were the additions of *Triatomaarthurneivai* Lenti & Martins, *Triatomajuazeirensis* Costa & Felix, *Triatomapetrocchiae* Pinto & Barreto and the subspecies *Triatomabrasiliensismacromelasoma* Galvão. Altogether, the collection currently has specimens captured in 15 North, Central and South American countries, including Brazil, which represents an increase of 23.7% in the number of countries compared to the year 2014 (Souza et al. 2014).

The structuring of Fiocruz/COLVET into four sections is recent. Until 2014 the collection had only whole specimens of triatomines deposited (Souza et al. 2014). As a consequence of Fiocruz increasing financial investment in institutional biological collections, Fiocruz/COLVET was able to increase its team of staff to sort, label and catalogue a backlog of specimens. Consequently, it was possible to include a large number of research voucher specimens: whole/semi-whole triatomine specimens, triatomine body parts (e.g., wings, genitalia, heads, legs, and abdomens), and/or DNA samples. The storage of these triatomine body parts and of DNA samples in the collection allows access to biological material used in diverse research areas, such as taxonomic and ecological studies (e.g., Ferreira et al. 2014; Silva et al. 2018; Rengifo-Correa et al. 2020; Gurgel-Gonçalves et al. 2021).

The main goal of Fiocruz/COLVET is to preserve and make the collection materials available for scientific research, conservation of biodiversity, and provision of services to the scientific community, entomological surveillance, and the general public. In this sense, it is extremely important that the collection is rich and diverse. Of the ~ 66 species of triatomines encountered in the Brazilian territory (Costa et al. 2021), 38 (57.6%) are present in the collection, which includes specimens from all biomes and all but three of the Brazilian states. The diversity represented by the species of scientific collections allows for a range of scientific possibilities beyond the specimen itself, as the metadata can be exploited, thus expanding the research power of the collection (Zaspel et al. 2020). Our results show how the states of Minas Gerais, Ceará, and Tocantins contribute to the composition of the collection, which is a consequence of well-structured partnerships between the Grupo Triatomíneos, Fiocruz/COLVET and institutions of the former states for the development of research over the past few years. It is also noteworthy that the most abundant species in the collection (*T.sordida*, *P.megistus*, *T.brasiliensis*, *R.nasutus*, *T.pseudomaculata*, *Rhodniusneglectus* Lent, and *T.vitticeps*) are, today, the most captured by entomological surveillance in Brazil (SVS 2019). This fact highlights the close partnership between Fiocruz/COLVET and the reference service in identification of triatomines.

Another practical example is the nine adult specimens of *T.costalimai*, captured in domestic and peri-domestic environments from Januária, in the north of Minas Gerais, which were sent to IRR/Fiocruz via the reference service and deposited in Fiocruz/COLVET (Souza et al. 2014). This species is endemic to the Cerrado and is associated with rocky environments, especially within limestone cracks and crevices (Schofield et al. 1980; Mello 1982). Although it is a little known vector, it has been frequently reported in domestic/peri-domestic environments of Goiás and Tocantins (Oliveira and Silva 2007; Machiner et al. 2012; Brito et al. 2017). The municipality of Januária and the north of Minas Gerais are transition areas between the Caatinga and Cerrado biomes, which is characterized by soil with limestone outcrops (Prous et al. 1984), the typical habitat of *T.costalimai*. Based on the ecological characteristics, the finding of this species in domestic and peri-domestic environments in this region suggests that the distribution range of *T.costalimai* includes the north of this state and, therefore, indicates the need for research and entomological surveillance in Minas Gerais for this species.

Important examples of the relevant partnership among Fiocruz/COLVET, entomological surveillance and research are the cases of ‘*R.nasutus*’ and *T.lenti* from the state of Minas Gerais and *P.lenti* from the state of Tocantins. It remains uncertain whether the specimens from domiciles in the municipality of Varzelândia (in the north of Minas Gerais) are *R.nasutus*. The analysis of morphological characteristics of the specimens is in agreement with the description of this species (Lent and Wygodzinsky 1979) as reported by Souza et al. (2014). *Rhodniusnasutus* is endemic to the semi-arid Caatinga of northeast Brazil, while *R.neglectus* occurs predominantly in open dry areas of the Cerrado. These two species occur in sympatry in the state of Bahia and, probably, in transition areas of the Caatinga-Cerrado (Lima et al. 2008; Abad-Franch et al. 2009) like the north of the state of Minas Gerais. Despite this, there is yet uncertainty about the limits of their distributions, mainly due to the morphological similarity between *R.nasutus* and *R.neglectus* and the chromatic variation of *Rhodnius* species in the Caatinga and in Caatinga-Cerrado transition zones (Pavan et al. 2015). Also, there is a report of *R.nasutus* collected in Ceará (in the northeast of Brazil) with a chromatic phenotype similar to that of *R.neglectus* (Dias et al. 2008). Therefore, it is yet to be determined whether the specimens from Minas Gerais morphologically identified as *R.nasutus* and deposited in the collection are in fact, *R.nasutus* occurring beyond the known limits of its distribution, or if they are *R.neglectus* with a chromatic phenotype similar to *R.nasutus*. In order to elucidate this question, molecular studies are being conducted with the specimens of ‘*R.nasutus*’ from the state of Minas Gerais.

*Triatomalenti* is distributed in Caatinga and seems to be more widespread in the dry forests of the Atlantic Forest in the Chapada Diamantina, in Bahia (Monteiro et al. 2018). Since 2006, *T.lenti* specimens from transitional areas of the Cerrado-Caatinga in the north of Minas Gerais have been deposited in the collection (Souza et al. 2014). The relatively frequent encounter of this species in the north of Minas Gerais during routine entomological surveillance suggests that species range limits may include transitional areas of the Cerrado-Caatinga.

*Panstrongyluslenti* is a species of the Cerrado, until now encountered in residences of the states of Bahia and Goiás (Patterson et al. 2009; Galvão 2014). Its natural ecotope is unknown, as well as the types of wild hosts with which it is associated. Also, so far, there are no reports of natural infection with *T.cruzi*, and, therefore, its role in the transmission of the parasite to humans is also unknown (Patterson et al. 2009; Georgieva et al. 2017). Fiocruz/COLVET has a single male specimen of *P.lenti*, which was found in a house in the municipality of Natividade in Tocantins in the year 2015 and sent by entomological surveillance workers to the collection for confirmation of the species identity. This is the first report of *P.lenti* in Tocantins, which opens the possibility of research about the ecology, distribution, and epidemiological importance of this species in this state. Similarly, the research voucher specimens relating to the first report of *Psammolestestertius* Lent & Jurberg in the state of Rio Grande do Norte (Silva et al. 2018) are deposited in the collection. Together, these deposited specimens underscore the importance of the collection for entomological surveillance, research, and preservation of recently reported specimens collected beyond their known distribution limits.

The specimens derived from laboratory stocks were deposited in the scientific repository when the Fiocruz/COLVET was first established in 1996. In contrast, the specimens of unknown provenance (i.e., without any associated information) have recently been deposited. The decision to deposit these specimens was because they are species rarely encountered/collected in sylvatic, domestic, or peri-domestic environments and, therefore, represent precious and rare materials.

Besides insects of the subfamily Triatominae, insects from other hemipteran families, included in the scientific repository are of great importance since these specimens can be used, for example, in further studies aimed at understanding the evolutionary processes within the order Hemiptera. In addition, the existence of the educational repository is also a strength of the Fiocruz/COLVET, as it is the basis for the vast majority of services provided by the collection to society. The large number of specimens held in this repository allows for the solicitation of training in identifying Triatominae found in all regions of Brazil. During training sessions, the health service workers (and other students) have the opportunity to handle many different triatomine species, which can sometimes result in specimens becoming damaged. However, due to the large number of specimens per species, the educational repository has sufficient material to replace any specimens damaged during training sessions. The large number of available specimens also allows the collection to offer training/courses to relatively large classes of ~ 15–20 people, without affecting the quality of teaching and learning through hands-on experience (as each student can have their own specimens). However, it is also possible to meet the demand for donations of specimens requested by students and professors, since the Grupo Triatomíneos at IRR/Fiocruz has a large insectary for the breeding of triatomines, which allows the replacement of specimens from the educational repository whenever necessary.

In 2013 the collection provided only one service. This can be explained by the fact that in that year, the official registration for service requests was initiated in the collection, using its own standardized form. In addition, in the first few years of registration (2013–2015), several requests were not registered in the forms of Fiocruz/COLVET because some activities were not considered as ‘services provided by the collection’. Thus, the number of services provided by Fiocruz/COLVET is probably much greater than that reported here, especially with regard to educational activities and the support of entomological surveillance (mainly training in the identification of Triatominae). In 2016, there was a higher number of services provided by the collection compared to other years. The depositing of biological material in the scientific repository was the service most provided by the collection in 2016. This material was derived from research projects and the reference service in identification of triatomines, which were already stored (before 2016) in the collection. However, due to insufficient staff, these samples have yet to be sorted, mounted, tagged, and catalogued.

Most services provided by the collection (41.7%) had the purpose of supporting educational activities, which included donations of specimens from the educational repository for use in classes and, in some cases, loaning specimens for science exhibition/fairs. This role of Fiocruz/COLVET in providing the lay public with contact with the specimens and, hence, with research is of great importance because it creates a connection and establishes a dialogue between academic institutions and wider society, which often has little or no direct contact with science (Bueno 2010).

The mission of Fiocruz/COLVET is to ensure the preservation of triatomines in the collection and the information associated with them, which are sources of strategic resources for the development of scientific research. Thus, not surprisingly, services to support research (35.7%) include Brazilian and non-Brazilian institutions, many of which have no established research partnerships with the collection. This demonstrates the recognition of the collection by the academic community and the importance of the collection to the development of scientific research nationally and internationally.

Services provided to support entomological surveillance accounted for the minority of all services provided by the collection. However, it is important to highlight that support for entomological surveillance, through the contribution to the taxonomic identification of triatomines, conducting training and donations of specimens, is fundamentally the duty of the reference service, which uses the collection to subsidize its activities. Therefore, the 38 (22.6%) services provided to support surveillance in the period analyzed represent a significant number of services requested to the collection.

Excellence in the provision of services by the collection is the primary way of the collection assisting the academic community, the Brazilian state/regional health departments, and society in general. The recognition and credibility that Fiocruz has both within the scientific community and civil society likely contributes to the total number of services requested to, provided by, Fiocruz/COLVET.

## ﻿Conclusions

The increase in the number of specimens and species from diverse regions of the Americas in the scientific repository Fiocruz/COLVET is a result of the financial investment of Fiocruz in organizing, managing, and maintaining the collections, and hiring and training personnel and curators of them. In addition to research support, the collection stands out for its support of educational activities and contributions to entomological surveillance and, therefore, to public health. The visibility of Fiocruz/COLVET in different sectors of Brazilian society, and the international community, demonstrates the important role of the collection for both scientific and civil community. The commitment of the curators of Fiocruz/COLVET is to maintain and expand the scientific and educational repositories, as well as ensuring continuity and excellence in providing services in future years.

## ﻿Financial support

Vice Presidência de Pesquisas e Coleções Biológicas da Fundação Oswaldo Cruz (VPPCB/Fiocruz); Instituto René Rachou/Fundação Oswaldo Cruz (IRR/Fiocruz); Conselho Nacional de Desenvolvimento Científico e Tecnológico (CNPq).

## Supplementary materials

Supplementary material 1 Figure S1 Data type schematic flowchart Explanation note Schematic flowchart showing the specimens of the Coleção de Vetores de Tripanosomatídeos (Fiocruz/COLVET) and the services provided by the scientific and educational repositories. Gray arrows indicate the origin and destination of specimens coming to the collection. Dotted arrows indicate the origin of specimens and the purpose of the service provided. *Indicates the reference service in taxonomic identification of triatomines of the Grupo de Pesquisa em Triatomíneos at Instituto René Rachou/Fiocruz. https://binary.pensoft.net/file/617444This dataset is made available under the Open Database License (http://opendatacommons.org/licenses/odbl/1.0/). The Open Database License (ODbL) is a license agreement intended to allow users to freely share, modify, and use this Dataset while maintaining this same freedom for others, provided that the original source and author(s) are credited.Raíssa Nogueira Brito

Supplementary material 2 Table S1 Data type list of the species Explanation note List of the species present in the educational repository of the Coleção de Vetores de Tripanosomatídeos (Fiocruz/COLVET). https://binary.pensoft.net/file/617445This dataset is made available under the Open Database License (http://opendatacommons.org/licenses/odbl/1.0/). The Open Database License (ODbL) is a license agreement intended to allow users to freely share, modify, and use this Dataset while maintaining this same freedom for others, provided that the original source and author(s) are credited.Raíssa Nogueira Brito
