# Supplementary figures and images for: ﻿Coleção de Vetores de Tripanosomatídeos (Fiocruz/COLVET) held at the institution Fiocruz Minas in Brazil: diversity of Triatominae (Hemiptera, Reduviidae) and relevance for research, education, and entomological surveillance
Source: Zookeys. 2021 Dec 1;1074:17–42. doi: 10.3897/zookeys.1074.69700 (PMC8654810; doi:10.3897/zookeys.1074.69700)

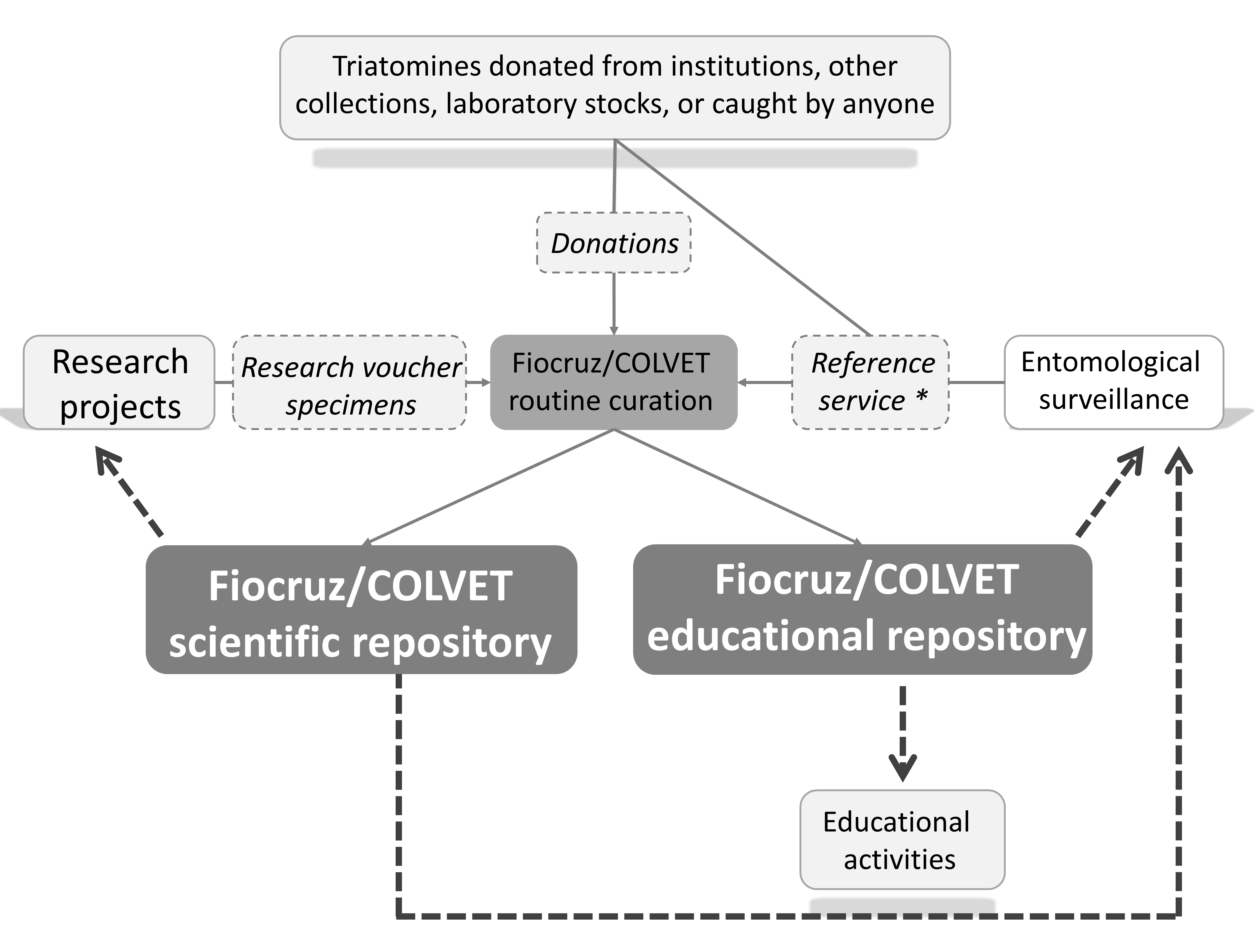

Supplement: Supplementary material 1 — Figure S1 [file zookeys-1074-017-s001.tif]
